# A Setup for Microscopic Studies of Ultrasounds Effects on Microliters Scale Samples: Analytical, Numerical and Experimental Characterization

**DOI:** 10.3390/pharmaceutics13060847

**Published:** 2021-06-08

**Authors:** Florian N. Gailliègue, Mindaugas Tamošiūnas, Franck M. André, Lluis M. Mir

**Affiliations:** 1 Institut Gustave Roussy, Metabolic and Systemic Aspects of the Oncogenesis (METSY), Université Paris-Saclay, CNRS, 94805 Villejuif, France; FLORIAN.GAILLIEGUE@gustaveroussy.fr (F.N.G.); franck.andre@cnrs.fr (F.M.A.); 2 Biophotonics Laboratory, Institute of Atomic Physics and Spectroscopy, University of Latvia, 19 Raina Blvd., LV-1586 Rīga, Latvia; mindaugas.tamosiunas@lu.lv

**Keywords:** ultrasounds, sonoporation, numerical simulations

## Abstract

Sonoporation is the process of cell membrane permeabilization, due to exposure to ultrasounds. There is a lack of consensus concerning the mechanisms of sonoporation: Understanding the mechanisms of sonoporation refines the choice of the ultrasonic parameters to be applied on the cells. Cells’ classical exposure systems to ultrasounds have several drawbacks, like the immersion of the cells in large volumes of liquid, the nonhomogeneous acoustic pressure in the large sample, and thus, the necessity for magnetic stirring to somehow homogenize the exposure of the cells. This article reports the development and characterization of a novel system allowing the exposure to ultrasounds of very small volumes and their observation under the microscope. The observation under a microscope imposes the exposure of cells and Giant Unilamellar Vesicles under an oblique incidence, as well as the very unusual presence of rigid walls limiting the sonicated volume. The advantages of this new setup are not only the use of a very small volume of cells culture medium/microbubbles (MB), but the presence of flat walls near the sonicated region that results in a more homogeneous ultrasonic pressure field, and thus, the control of the focal distance and the real exposure time. The setup presented here comprises the ability to survey the geometrical and dynamical aspects of the exposure of cells and MB to ultrasounds, if an ultrafast camera is used. Indeed, the setup thus fulfills all the requirements to apply ultrasounds conveniently, for accurate mechanistic experiments under an inverted fluorescence microscope, and it could have interesting applications in photoacoustic research.

## 1. Introduction

Ultrasound (US) waves interact with cell membranes. Under specific conditions, they can even permeabilize the cells. This phenomenon is known as sonoporation. It can either be achieved with US alone [1] or with contrast agents, such as gas microbubbles (MB), which will potentiate the ultrasounds by cavitating [2,3].

The previous studies in the field of sonoporation have analyzed the influence of US output parameters (frequency, number of cycles, duty cycle, and acoustic pressure) through the sonoporation of cells in suspension in the presence of MB [4,5,6,7].

The exact sonoporation mechanisms are still under investigation. It is thought that the mechanisms of the US-induced sonoporation are linked to microbubbles oscillations. Equation (1) is an approximation to determine the resonance frequency of the MB oscillations linked to MB radius, described in the literature [8]:

ω_0_^2^ = (1/ρ R_0_^2^) [3γ (P_0_ + 2σ/R_0_ + 2χ/R_0_) − 2σ/R_0_ − 6χ/R_0_], (1)

with ω**_0_** the resonance pulsation, ρ the liquid density, R**_0_** the initial bubble radius, **γ** the polytropic gas constant, P**_0_** the hydrostatic pressure, σ the interfacial tension coefficient and χ the elasticity modulus of the lipid shell.

MB vibrates under different oscillation regimens depending on the mechanical index, defined as the peak negative pressure (in kPa) divided by the square root of the frequency (in Hz), as seen in Equation (2):

MI = PNP/f^1/2^, (2)

Depending on the mechanical indexes, MB will oscillate and follow different cavitation regimes. These will lead, among others, to cell massage, microstreaming, or microjets, which are the physical mechanisms inducing the sonoporation [9]. Several parameters are implied in the efficiency of single-cell sonoporation, the most important being related to the dynamic behavior of MB (displacement of the MB towards the cell or in the opposite direction), MB radius (R); MB to cell distance (D); D/R ratio, MB and cell concentrations, and proportions and MB clusterization [4,5,6,10,11,12].

To precisely understand the effects of US and MB on cell membranes, specific setups need to be designed. Indeed, classical bulk exposure of cells to US and MB is usually performed in large water baths [6,7,13,14]. While this exposure system allows these experiments to be easily performed, it has severe limitations. For example, the ultrasonic beam is emitted towards the center of the bath. Thus, only a small part of cells and MB are exposed to ultrasounds at a given time. To prevent a huge heterogeneity in cell exposure, magnetic stirring is often used as a solution to diminish this heterogeneity, but it has several drawbacks. First magnetic stirring can disturb streams inside the fluid. Then there is no individual cell control of the time passed under a given pressure or of the average power to which each individual cell is exposed. Finally, electromagnetic fields are known for their potential ability to interact with cells [15]. Therefore, it seemed very important to find experimental conditions avoiding stirring of the solution, thus, avoiding potentially undesirable and synergetic interactions between fluid mechanical agitation, electromagnetic fields presence, and ultrasound pressure. An improved setup should allow the strict control of all the parameters affecting the cells, and thus, the addition of uncharacterized effects of the electromagnetic fields and stirring is not an acceptable option. On the contrary, another option that would be desirable in a new setup would be the possibility to perform real-time microscopic observations of the US and MB interactions with the cell [3,4,5,16,17]. This is often achieved using a special chamber (ultrasound compatible and optically transparent) placed in a water bath: The OptiCell (Nunc, Thermo Fisher Scientific, Wiesbaden, Germany) or the Clinicell™ (MABIO, Tourcoing, France) [4,18,19]. Working with neither flat nor rigid surfaces, however, made it difficult to precisely know the ultrasonic field pressure inside the Clinicell™ because of the deformability of the Clinicell™ membrane. The characterization of the acoustic pressure in these chambers has only been determined very recently [20]. These chambers also require the use of about 10 mL of sample. A system that enables homogenous sonoporation with real-time observations of microliters samples under the microscope explains the development of the setup reported in this article.

We thus describe here a novel setup to expose cells to US, either in suspension or attached, in the presence or the absence of MB, allowing real-time follow-up in fluorescence or bright light microscope. Numerical simulations were performed to make an optimized design of the setup, as well as to characterize it. First experimental observations were performed using animal cells and Giant Unilamellar lipidic Vesicles (GUVs) that allowed the real-time detection of the sonoporation.

## 2. Materials and Methods

Materials: DOPC (1,2-dioleoyl-sn-glycero-3-phosphocholine), DLPC (1,2-dilinoleoyl-sn-glycero-3-phosphocholine) and POPC (1-palmitoyl-2-oleoyl-sn-glycero-3-phosphocholine) were purchased from Avanti Polar Lipids (Alabaster, AL, USA). All other chemicals and solvents were purchased from Sigma Aldrich (St. Louis, MO, USA). All reagents were used without further purification. The purity of DOPC and DLPC was checked by mass spectrometry before use, to avoid employing source materials that would already be partially oxidized. Commercial solutions of microbubbles (Bracco, Milan, Italy) were suspended in 0.9% NaCl.

Simulations: Models of the setup design were created with the COMSOL Multiphysics version 5.2 (COMSOL AB, Burlington, MA, USA) to define the more appropriate geometries fulfilling the requirements listed in the Introduction section. We simulated the normal acceleration of a boundary that generates ultrasonic waves. These waves went through the desired setup. The fundamental parameters of each material were used, such as density, the speed of sound in the material, and its specific attenuation coefficient. From these parameters and the input field, we simulated acoustic pressure at any point of the setup. Calculus and equation solving were done with Maple V release 4.00a (Maple Inc., Waterloo, ON, Canada).

Electroformation Protocol: Giant Unilamellar Vesicles (GUVs) are a convenient model for studying cell membranes. They are lipid bilayers, filled and surrounded by an aqueous medium saline or sucrose buffer even though they are slightly less rigid than the cells. The lipid composition can be chosen to fit the one of the membranes. Most importantly, their lipids are easy to extract and analyze by mass spectrometry as there are no organelles or cytoplasmic compounds to interfere during the analysis. The vesicles were prepared using an electroformation protocol derived from classical procedures [21,22]. The formation was conducted at 4 °C to avoid any lipid degradation, due to temperature, to ensure consistency between the various experiments, as well as to consider the phase transition temperature of the lipids, and thus, preparing all the vesicles in the fluid state. The desired lipid was dissolved in chloroform at a concentration of 1 µg/µL to which 1% DOPE Rhodamine was added. Then, 15 µL of the lipid solution were deposited on the conducting side of two glass slides coated with indium tin oxide (Sigma Aldrich, St. Louis, MO, USA); the slides were thoroughly washed with ethanol, then with distilled water, and then then ethanol and dried before use). The slides were then kept under vacuum for 2 h in a desiccator to remove all traces of organic solvent. A chamber was assembled with the slides spaced by a 1.5 mm silicone isolator (Sigma Aldrich, St. Louis, MO, USA). The chamber was filled with a buffer solution (240 mM sucrose, 1 mM NaCl, 1 mM KH_2_PO_4_/K_2_HPO_4_, and pH 7.4). The electroformation was conducted in a low conductive medium containing a minimal number of salts to avoid any interference in the mass spectrometry analysis, due to salt adducts formation (for example, lipids complexed with sodium). The slides were connected to a function/arbitrary waveform generator (HP Agilent 33120A), and a sinusoidal AC field with a voltage of 2 V peak to peak, and 10 Hz was applied and maintained under these conditions for 12 h. Finally, a square-wave AC field of 3 V peak to peak was applied at 5 Hz for 1 h to detach the GUVs from the slides. The total formation time was therefore 13 h.

Cell culture: Chinese hamster ovary (CHO) cells were cultured in DMEM (Sigma–Aldrich, St. Louis, MO, USA) supplemented with 10% fetal bovine serum, 1% penicillin, 1% streptomycin (Life Technologies, Cergy-Pontoise, France), at 37 °C and 5% CO_2_ humidified atmosphere. After trypsinization, the cell suspension was diluted to 10^5^ cells/mL, then 100 μL volume was deposited on the microscope cover slide (18 × 18 mm) and stored in the incubator for 4 h for the attachment of the cells. During the last 30 min of the cell attachment period, the cells were incubated with calcein-AM (Sigma Aldrich, St. Louis, MO, USA) at 10 μM final concentration.

Before cell sonoporation and imaging, the slides were washed with 1× PBS (Lonza, Rockland, ME, USA) and attached on the portable setup with 3 to 25 cells visible in the microscope field of view.

**Figure 1:**
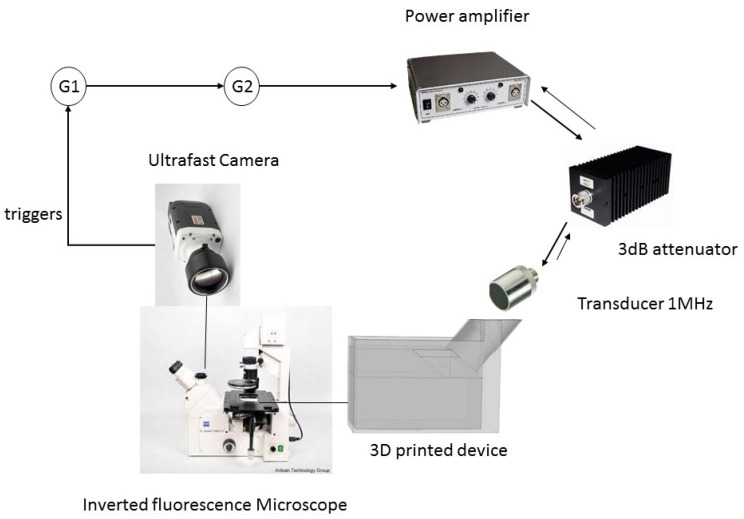
Two generators, one master, G1 (Agilent Technologies, Santa Clara, CA, USA), and one slave, G2 (Rigol Technologies, Beijing, China), were used to trigger and generate a 1MHz sinusoidal signal with chosen pulse repetition frequency, duty cycle, and amplitude. A power amplifier AAP0.5-6-200 (ADECE, Veigné, France) set to its maximum gain amplified the signal. A 100 W, 3 dB power attenuator was added to the output of the amplifier to protect it from reflections, due to potential impedance mismatch with the ultrasonic transducer. The 1 MHz immersion transducer ICFM016 (Sofranel, Sartrouville, France) powered by the generators and amplifier emitted the ultrasounds: Sinusoidal signal with chosen pulse repetition frequency, duty cycle, and amplitude.

Ultrasound exposure material and protocol: The ultrasound exposure material is shown in Figure 1. The chamber containing the cells or the GUVs, which results from the numerical simulations and analysis, is described in the results section. In cell sonoporation experiments, 30 μL of 1× PBS were added to the exposure chamber in control and US exposed groups. Sonovue microbubbles (Bracco Diagnostics, Geneva, Switzerland) were prepared according to the manufacturer’s instructions and diluted 100 times in 1× PBS, yielding a concentration of 1.36 × 10^5^ MB/mL, counted in a hematocytometer. 30 μL of diluted MB suspension was used as the cell incubation media in the US + MB group. Each experimental group was replicated three times. The fluorescence of a total of 221 CHO cells was examined. 

The GUVs sonoporation mix was a 10 µL solution composed of 1/3 DLPC GUVs solution with 2% DOPE Rhodamine, 1/3 MB solution (10:1 MB to cell ratio), 1/3 of a 240 mM sucrose buffer with 2 mM Lucifer Yellow.

Image acquisition: Observations were realized with an inverted fluorescence microscope Axiovert100 (Carl Zeiss, Oberkochen, Germany) placed either an HMK Zeiss camera or an ultrafast Phantom Miro m310 camera (Vision Research, Wayne, NJ, USA). The conditions for the ultrafast image acquisition were 990 μs exposure time per frame, 1000 fps frame rate, 128 × 128 pixel resolution, and 8 bit vertical depth. Depending on fluorescence excitation and emission properties, filter set #10 (FITC) was used for LY or calcein-AM fluorescence detection and filter set #15 (TRITC) for DOPE Rhodamine. The fluorescence microscopy images were preprocessed with open-source Fiji software. The MB motion history images were created by using the Fiji TrackMate plugin [23].

System calibration: The system calibration was realized at Inserm U910, Tours, France, using a 0.2 mm needle hydrophone (Precision Acoustics, Dorchester, UK). Calibration was performed along the z-axis, then along the x-axis at the focal spot. The input voltage-output pressure response was evaluated, and the bandwidth characterization of the transducer was characterized.

Statistical Analysis: Statistical analysis was conducted with the software Prism 7.02 (GraphPad, San Diego, CA, USA). Because of the number and the nature of the replicates, the unpaired nonparametric Mann-Whitney test was used. Results were considered statistically significant if the p-value was under 0.05 (95% confidence): *p* < 0.0001∶ ****, *p* < 0.001∶ ***, *p* < 0.01∶ ** and *p* < 0.05: *.

## 3. Results

### 3.1. Development of a Model to Predict the Ultrasonic Field in a Small Medium Volume Encompassed by Rigid Walls

The objective of the new applicator was the controlled sonoporation of GUVs and cells in aqueous media. Therefore, an immersion transducer was used to reproduce biological conditions. Working with an immersion transducer also implies complete immersion of the transducer tip into an aqueous medium. Ultrasounds obey laws for reflection and transmission at an interface. Each medium is characterized by its specific acoustic impedance. Its absolute value, r, can be defined as in Equation (3):

r = ρ c,(3)

where ρ is the medium density and c is the speed of sound in the medium. The transmission of ultrasounds from one fluid to another is affected by the difference in impedance between the two media. Solid materials on the ultrasonic pathway can be considered as fluid saturated porous materials [24]. Two types of elastic waves propagate through solids: The longitudinal one and the shear one. Depending on the dimensions of the solid compared to the wavelength, the solid will react differently to pressure waves. Thus, the speed of the longitudinal waves in the material will vary. For extended solids, the bulk speed is the one to consider. The bulk and shear moduli of the solid, as well as the density, are required to determine the bulk speed. For smaller ones, like the slides present in this article, the bar speed is to be preferred. For the bar speed, of longitudinal waves, the Young modulus is necessary. Each modulus describes the material’s response (strain) to a specific kind of stress: The bulk modulus describes the response to isostatic compression, the shear modulus describes the response to shear, and Young’s modulus describes the response to linear stress.

Under normal incidence, the intensity transmission and reflection coefficients are represented by Equation (4):

T_i_ = 4 r_2_ r_1_/(r_2_ + r_1_)^2^ and R_i_ = (r_1_ − r_2_)^2^/(r_1_ + r_2_)^2^, (4)

Reflection coefficients at each interface are deduced using the Equation (5):

R_i_ + T_i_ = 1, (5)

Modeling the US pathway and calculating the transmission and reflection coefficients allowed selecting the most appropriate materials. It is important to note that only if the two impedances of the materials are identical (r_1_ = r_2_), then T_i_ = 1. The near acoustic pressure field of the transducer is a highly heterogeneous region. After the last zero pressure, the field reaches a maximum. That maximum is called the focal spot. The distance between the transducer and the focal spot is called the focal distance, which is 6 cm with the transducer used here. Thus, the transducer was fixed in a 6 cm long sealed plastic to avoid exposing GUVs or cells in near field inhomogeneous conditions. For the proposed setup (Table 1) a large, predetermined water volume (medium 1 of acoustic impedance r_1_) is necessary for the transducer immersion. A polystyrene or glass layer (medium 2 of acoustic impedance r_2_) placed at the bottom of the cylinder separates liquid medium 1 from liquid medium 3 in which the cells are located. If the cells are attached to a substrate, they are at the fixed position z = e_1_ in a small volume of medium 3, if GUVs or cells in suspension are used, they float in a small volume of physiological liquid (medium 3 of acoustic impedance r_3_) limited by medium 2 and medium 4 (medium 4 of acoustic impedance r_4_) made of polystyrene or glass. This small volume ensures that the cells are located near the focal spot. The reasons for using such materials are two-fold. The most important characteristic of these materials is transparency to ultrasounds and light. Advantages of glass are it is more optically transparency and has better durability, because it does not suffer from scratches. The ultrasonic beam ultimately transmits to air (medium 5 of acoustic impedance r_5_). A schematic representation of the acoustic wave propagating path is described in detail in Appendix A.

### 3.2. Practical Design of the New Applicator

#### 3.2.1. Transducer Type

**Figure 2:**
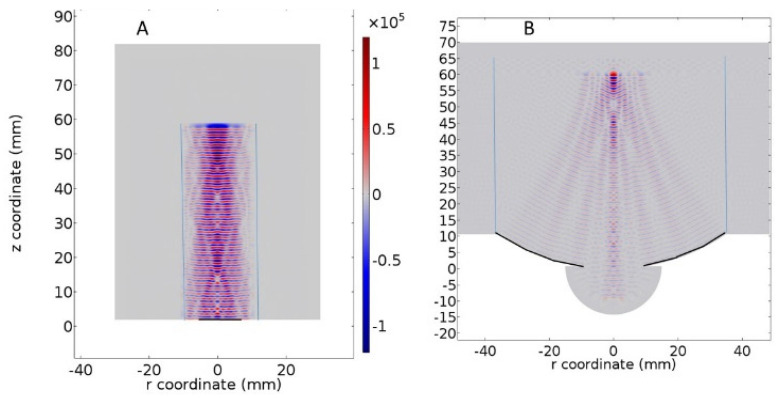
Simulated ultrasonic field of a flat transducer (**A**) or a focused transducer (**B**). The focal spot for the focalized transducer is narrower, but the pressure generated is more intense. The vibrating surface elements of the transducer are depicted in dark bold. The polyethylene walls of the tubes are blue. The focused transducer model was adapted from the COMSOL acoustic modulus library.

Transducers can be either flat or focused. In both cases, the ultrasonic beam focuses. The difference lies in the focal spot characteristics. A focused transducer will have a shorter focal distance, a narrower focal spot, and a more intense pressure at the focal spot, as well as a more precise spatial resolution. Simulations were run with both types of transducers in a sealed cylinder. The simulated focused transducer was adapted to have the same focal distance as the flat transducer. The main results are shown in Table 2. The focal spot is less homogenous with a focused transducer. The Isosurfaces are defined as the surfaces in which the pressure is identical ± xx% (Table 2). The flat transducer presented a better homogeneity in pressure amplitude which appeared to be more suitable for a mechanistic study (Figure 2). Therefore, the numerical and experimental results reported below were all achieved with a flat transducer. This allows a larger focal spot and a more homogenous field.

#### 3.2.2. Constraints of Simultaneous Exposure to Light and US: Modelling US Nonnormal Application

For practical reasons, phase contrast generation under the microscope requires the presence of a condenser, and light normal incidence corresponds to the most often used geometry [25,26]. The problem with ultrasounds application under a normal incidence is that it forces the transducer to be directly above the sample, making it impossible to illuminate the sample with bright light under the microscope as there is no space for both the transducer and the microscope condenser above the exposure device. This makes the use of microscope phase contrast almost impossible. Thus, normal ultrasounds incidence cannot be applied. The transducer being in a sealed water cylinder, the transducer emits ultrasound towards the sample at a precise angle. The setup developed in the present study enables light illumination under unmodified phase contrast. However, modeling the US beams hitting the targets under an oblique incidence is a complex issue. To this end, a modified version of the Snell-Descartes law of refraction known in optics, c_2_ sinθ_i_ = c_1_ sinθ_t_, was used here for acoustics. There is a critical angle above which there is an only reflection and no transmission: It is given by sinθ_c_ = c_1_ c_2_. As long as θ_i_ < θ_c_ there is transmission, and it is possible to estimate the power transmission coefficient T_π_, following Equation (6).

T_π_ = 4 (r_2_/r_1_) (cosθ_t_/cosθ_i_) / (r_2_/r_1_ + cosθ_t_/cosθ_i_)^2^ (6)

**Figure 3:**
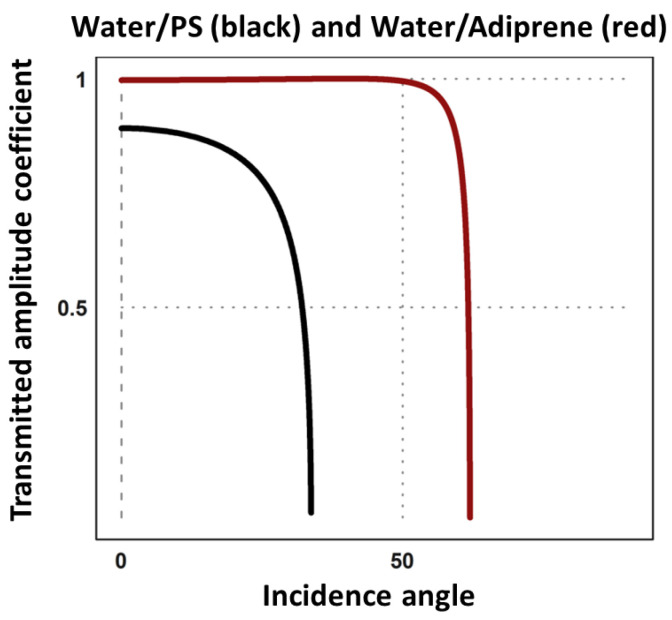
Transmitted amplitude coefficient for oblique incidence depending on the angle to the normal. The black line represents the transmitted amplitude coefficient for a water/polystyrene interface. The red line represents the transmitted amplitude coefficient for a water/adiprene interface for which both a critical angle and an intromission angle exist. The transmitted amplitude coefficient above a critical angle is not defined. For such angles, there is a total reflection of the longitudinal waves. Considering the described setup, the transducer’s intromission angle was fixed at 26.5°.

Simulations corroborate the existence of the critical angle. The power transmission coefficient T_π_ is represented in Figure 3 for water/polystyrene interface and water/adiprene.

For angles above the critical angle, the pressure is attenuated along the z-axis before the interface. That can be explained by the total reflection of the ultrasounds on the interface and mostly by destructive interferences with the original beam. The material chosen has a major impact on the fraction of the incident power transmitted to the sample. Thus, materials must be chosen carefully. Ideally, the optimal medium for the simultaneous exposure to ultrasounds and observation under the microscope would be an optically transparent material with an acoustic impedance like the one of water. That would ensure that we could observe the sample and have maximum transmitted power. Some energy is still transmitted from the first medium to the second, because the shear waves are still transmitted at these angles. Shear waves having a lower propagation speed, the acoustic impedances of the materials for shear waves change. And a second critical angle greater than the one for longitudinal waves exists. Thus, for the setup, described here below with glass slides, there are waves transmitted to the sample even for flatter angles.

#### 3.2.3. Other Constraints and Device Fabrication

**Figure 4:**
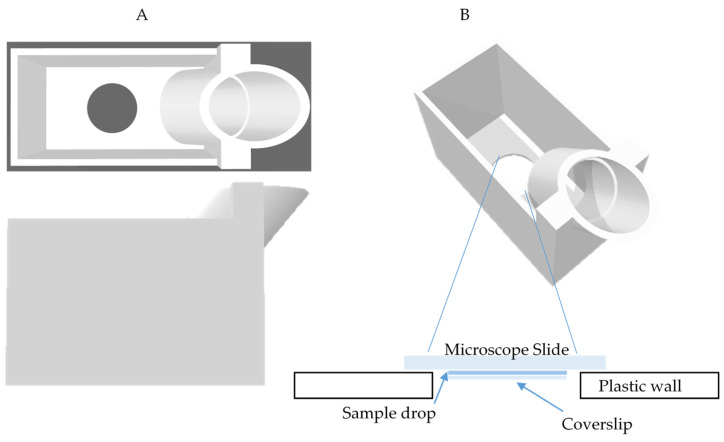
Image of the 3D printed setup for the simultaneous exposure and observation under the microscope of a small liquid volume. (**A**) Top and side view of our system. (**B**) Zoom on the bottom part of the device (schematic view). The sample is placed under the circular coverslip visible at the bottom of the device and the microscope slide. The transducer is fixed in the inclined upper cylinder.

Under oblique incidence, the water weight in the cylinder creates instability. That imbalance causes the cylinder to fall over because of its own weight. To stabilize the system, the base was widened, and the whole device was 3D printed (iMaterialise, Leuven, Belgium) for waterproofness and alignment precision. A 1.2 mm thick microscope slide was placed on its bottom, and it was sealed with silicon (Figure 4).

#### 3.2.4. Sample Positioning

The sample is placed at the transducer’s focal distance trapped between the microscope slide and a circular coverslip. This enables a low volume to be used in each experiment and ensures that the microbubbles and the GUVs or cells are in the same plane, which is advantageous for the simultaneous observation and exposure to the ultrasounds. A hole was designed at the focal spot of the transducer, and on this hole, either the slide microscope or a polystyrene film can be placed. As described in the simulation part, the materials constituting the slide and the coverslip play a major role in the acoustic pressure along the wave propagation axis. Between the two glass slides, interferences can occur when the incident and reflected beams meet. Depending on the phase of the two waves, the resulting power amplitude will vary. In our experimental setup, the distance between the two glasses slides depends on the volume of the liquid sample trapped between the slides. It typically varies from 10 to 20 µm. As far as absorption is concerned, the glass of the microscope slide was the most absorbing medium on the acoustic path. However, attenuation (including the effect of absorption) was responsible for approximately 5% loss of acoustic power if the glass layer is e1 (1.2 mm) thick. Even with a hypothetical 10 mm thick glass slide, attenuation represents no more than a 5% loss of the acoustic power (Appendix A.2). It should be noted that while attenuation is almost negligible in the model as the distances are rather small, it is still considered to ensure more reliable results. The pressure amplitude is a function depending, amongst others, on the distance between the two slides noted L. The phase of the reflected wave will determine the interference pattern between the incident and reflected waves. Simulations reveal (Appendix A.3) that the pressure amplitude can be either amplified or reduced. The distance between the two slides is calculated by dividing the volume of the drop and the surface of the cover slide. Therefore, it is variable with changes in volume. Glass was still ultimately chosen because its flatness, rigidity, and resistance to scratches guarantee better optical transparency.

### 3.3. Device Experimental Calibrations

Several parameters were measured: The pressure amplitude along the wave propagation axis (z-axis), the pressure amplitude at the focal distance along the x-axis (orthogonal to the z-axis), and the US pressure output dependency on the input tension of the generator. It should be noted that all the parameters of the calibration, but the last one, were tested in a water tank both with and without our 3D printed setup to see how the proximity of rigid walls might modify the ultrasonic field. This enabled the better fitting of the simulations to the observed reality. For example, the ultrasonic pressure at the level of the cells cannot simply be measured with a hydrophone as the region of interest is a thin layer compressed between two other compartments possessing rigid boundaries (the glass slide and the microslide), thus, there is no open access to needle hydrophones which diameter is larger than 20 µm and so fragile that contact with solid surfaces must be avoided.

Therefore, simulations are the only way to reach an acceptable approximated value of the acoustic field applied. In conditions of a transducer emitting in a free water volume, the measured value of the peak pressure amplitude 500 kPa at the focal spot. Thus, simulation parameters were adapted to find the same value. The addition of the setup in the simulations estimates the loss of power, due to the glass layer as in the focal spot, the pressure was limited to 380 kPa.

### 3.4. Experimental Results Using the New Setup

**Figure 5:**
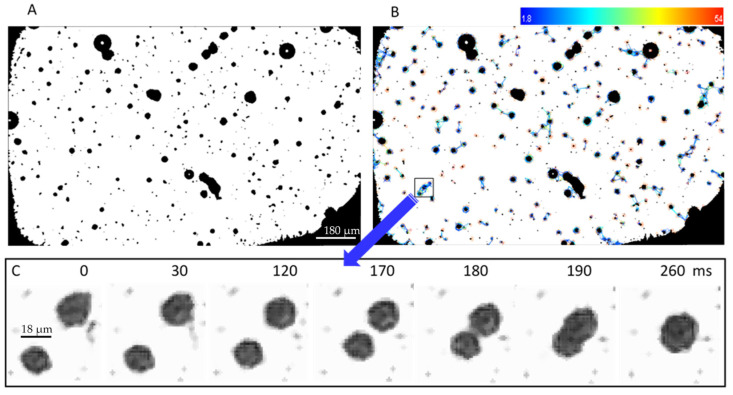
Microbubbles dynamics over time displayed in a motion history image. (**A**) Original MB image extracted from video data; (**B**) image analysis using the Fiji TrackMate interface—the color scale indicates track displacement in arbitrary units; (**C**) one example of MB trajectory visualization: Frames #1−#260 show coalescence of two MB clusters (known as “bubble grapes”).

One of the main issues was the assessment that sufficient acoustic pressures were reached at the focal spot, qualitatively validating the calculations reported here. The traditional needle hydrophone measurements were not possible, due to the tens of micrometers thickness of the sealed setup. Thus, the local pressure field was experimentally characterized based on microbubbles response to ultrasounds, recorded using a Phantom Miro 310 ultrafast camera (Figure 5). Indeed, the MB trajectories (that the presented device allows visualization) are related to the acoustic pressure within medium 3, containing the MB. For further details, see the Appendix A.1 for a five-layer model of ultrasonic wave transmission and reflection.

During US application time of 0.8124 s, the microbubbles oscillatory response was detected within the whole ROI (Figure 5B). The MB motion (caused by the Bjerknes forces) provided the implicit determination of acoustic pressure generation and distribution. We evidenced the generation of acoustic radiation forces, causing MB clusters to migrate and associate in the acoustic field (Figure 5C), single MB to attract (or repel) each other, or to gather at certain points within ROI during insonication, or to execute erratic motions, referred to as “dancing” behavior [27]. Video file is available as Supplementary Material  (990 μs exposure time per frame, 1000 fps frame rate, 768 × 480 pixel resolution, 0.37 s duration).

**Figure 6:**
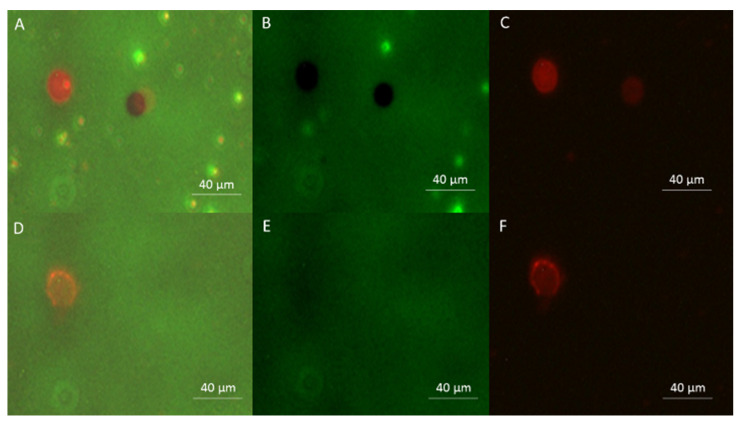
Fluorescence microscopy imaging of GUVs. (**A**) Superposition of LY and Rhodamine imaging before ultrasounds delivery (**B**) Fluorescence imaging of the Lucifer Yellow present on the outside of the GUVs before ultrasounds delivery. (**C**) Fluorescence imaging of the Rhodamine present in the GUV membrane before ultrasounds delivery. (**D**) Superposition of LY and Rhodamine imaging after ultrasounds delivery. (**E**) Fluorescence imaging of the Lucifer Yellow present on the GUVs after ultrasounds delivery. (**F**) Fluorescence imaging of the Rhodamine present in the GUV membrane after ultrasounds delivery. It is important to note that of the two GUVs in panels A, B, and C (before US delivery), only one GUV remains in panels D, E, and F (after US delivery) and was permeabilized, while the second GUV moved away. Also important is the presence of MB observable under Lucifer Yellow fluorescence before the US application (panels A and B). Their absence in panels D and E confirms that US were delivered, and that MB disintegrated after their inertial cavitation at the high MI used in the experiments.

To prove that sonoporation can indeed be performed in the new setup, a test was conducted to observe GUVs permeabilization after exposure to ultrasounds: A 10 µL drop of a mixture of Rhodamine labeled GUVs and MB in a Lucifer Yellow (LY) containing medium was placed on the microscope slide and covered with a cover slide. The upper chamber of the system was filled with water, and the setup was placed under the microscope. Microbubbles were observed under phase contrast. The Rhodamine red fluorescence of the GUVs membrane allowed GUVs detection under fluorescence microscopy. GUVs permeability was assessed by the LY green fluorescence penetration in the GUVs. Indeed, impermeable GUVs appear as black discs under LY fluorescence imaging, because when the GUVs are not yet permeabilized, they do not let the LY present in the outer medium to enter them, which appear as black spots in the fluorescence background, due to the LY presence in the external medium. If a GUV becomes permeable, the LY fills it by diffusion through the membrane, and GUVs detected by the Rhodamine fluorescence become undetectable in LY fluorescence imaging (Figure 6 panel E).

Using the proposed setup, high intensity ultrasounds (380 kPa, 40% duty cycle, and 10 kHz pulse repetition frequency) were delivered. Under these sonication conditions, the microbubbles cavitate and explode in several cycles: In less than 2 s, no microbubble was found in the solution anymore [28]. Thus, at this pressure, long sonication times are not necessary to study the US/MB interaction. Intact GUVs can be detected on both fluorescence channels, whereas permeabilized GUVs are only detectable in the Rhodamine fluorescence channel. These images demonstrate that sonoporation can be performed in a small compartment limited by two rigid walls and is detectable under fluorescence microscopy using the novel setup.

**Figure 7:**
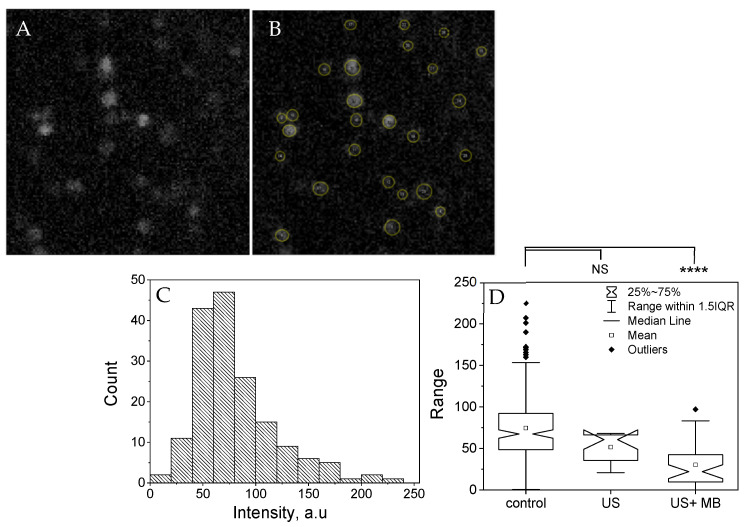
Sonoporation of cells insonicated in the setup. (**A**) An example of the acquired ultrafast images; (**B**) segmentation of cells within the fluorescence microscopy image; (**C**) Calcein-AM fluorescence distribution in the control cell group; (**D**) Notched boxplots compares the fluorescence intensity distribution of the control and the two experimental groups (US; US + MB); **** corresponds to *p* < 0.0001.

Figure 7 displays the demonstration of the sonoporation of the CHO cells preloaded with calcein-AM. An example of the acquired ultrafast images is shown in panel A, while the result of the fluorescent cells’ manual segmentation is shown in panel B. Each cell’s mean fluorescence was evaluated separately within the segmented pixels. Calcein-AM fluorescence distribution in the control cell group (183 cells in total, no US nor MB) is represented by the histogram (C) showing the fluorescence intensity Gaussian-like distribution pattern. The notched boxplot (D) compares the fluorescence intensity distribution of the control and the two experimental groups—17 cells with US applied for 0.8124 s; and 21 cells insonicated for 0.8124 s in the presence of MB (US + MB group). Since the notches in the boxplots do not overlap, the medians between control cells and sonoporated cells (US + MB group) differ with 95% confidence. Statistical analysis confirmed that at 0.0001 level, the difference of the fluorescence means between control and sonoporated cells was highly significant. This suggests that the exposure to US in the presence of the MB resulted in the loss of a fraction of the calcein-AM molecules from CHO cells, and thus, that US were successfully applied to provoke cell sonoporation using the portable setup described in this article.

## 4. Discussion

A specific system has been developed that enables the successful observation of biological objects and their exposure to ultrasounds. It is particularly well suited for samples produced in small amounts like GUVs. Indeed, GUVs are electroformed in low concentrations and small quantities. This limited production imposes the use of minimal working volumes to detect still the GUVs under the microscope. Until now, cells were often exposed in a Clinicell™, which is a sterile bag of about 12.5 mL internal volume, or in Opticell™ (discontinued by manufacturer) [4,25,29]: Their use would impose a very high GUVs dilution factor (>10). GUVs cannot be diluted by a factor over ten as their concentration in the final solution would be too low to detect the GUVs under the microscope. If both the GUVs and transducer are in an aqueous bath, the minimum volume used would be at least a water cylinder volume of V = π R^2^ H. With a radius R of, at least, the external radius of the transducer (12.5 mm) and the height H of at least the focal distance (6 cm), the minimum volume is 30 mL. Classical means for GUVs electroformation protocol takes 13 h and produces not more than 1.5 mL of GUVs solution at relatively low concentration. Thus, production, even slightly diluted, would only suffice for one or two experiments per day. However, GUVs production is not only time-consuming, but moreover, purified lipids are expensive, and multiplying GUVs production would have a major cost. Moreover, the Clinicell™ membrane is not a flat nor rigid surface (Table 1), so the ultrasonic beam can be disturbed. In our proposed setup, 10–30 µL of GUV or cells solution is sufficient. Therefore, GUV concentration can remain very high. GUVs experiments are then easy to perform and reproducible because several replicates can be done with the same batch. While GUVs are not perfect cell models because their membrane is less rigid than the membrane of the cells, GUVs are easier to approach from a mechanical point of view as they are much simpler.

The setup is also very interesting in the case of cells either in suspension or attached. Indeed, three distinct possibilities exist to study cell sonoporation: Cells in suspension in a large volume of cell culture medium, cells in Clinicell™ intermediate volume and neither flat nor rigid walls, or the presented setup with a small volume and flat and rigid walls for better control of the ultrasonic pathway. This enables sonoporation to be performed in the presence of expensive molecules of interest or in special media, since only a very small fraction (10 µL) of medium will be used.

Another obvious advantage of this setup is the continuous monitoring of the sonoporation, under the microscope. This allows better control of the sonoporation parameters and facilitates analyses if a fast camera allows video recording of the events occurring during the exposure to the US [10,30]. For example, not only fluorescence kinetic analysis can be performed, but moreover, if a GUV or a cell becomes permeable, it can be verified afterward if there was a microbubble in its vicinity and what was the trajectory of the MB. In previous studies, it was found that MB displacement away from the cell likely facilitates cell reversible sonoporation and preserves cell viability, while nondisplaced MB or MB displaced towards the cell surface cause a greater number of cells to die [10,11]. Moreover, experiments can be stopped as soon as microbubbles implosion and disappearance are visually detected. As a consequence, total exposure time could be inferior and more precisely controlled compared to the one or two minutes exposures classically found in the literature, preserving cell viability.

The setup is also applicable to study the time averaged translational behavior of MB, to support the theoretical calculations for microbubble attraction or repulsion events depending on the sign of the Bjerknes force.

**Figure A1:**
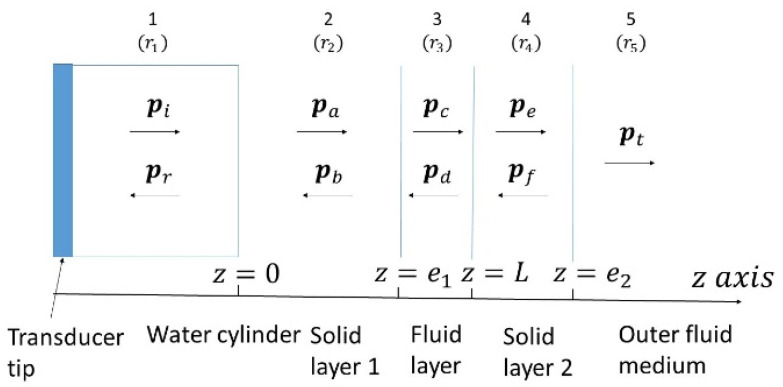
Schematic representation of an ultrasonic wave transmission and reflection in five layers adapted from the three layer problem described in [34]. If GUVs or cells are used, they are located in medium 3: The GUVs in suspension, and the cells either attached at z = e1, or z = L, or in suspension.

**Figure A2:**
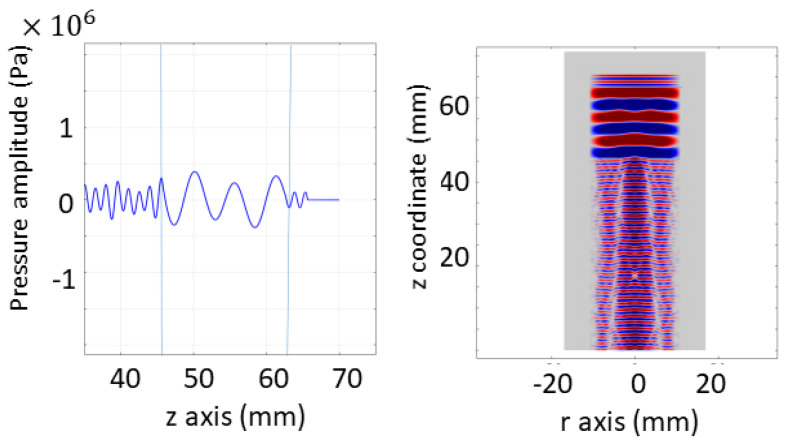
Simulated pressure amplitude for a 20 mm thick glass slide. The blue bars represent the glass slide edges. A 5% amplitude drop is expected for this thickness. No clear difference inside the glass is observed, which is coherent with attenuation by absorption being almost negligible.

**Figure A3:**
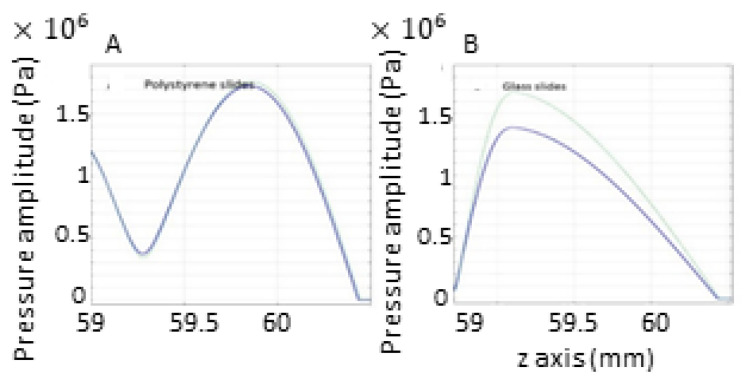
Absolute pressure amplitude along the z-axis depending on the distance between the slides (10 µm is the dark blue curve or 20 µm is the light blue curve). (**A**) Absolute pressure when the two slides are in polystyrene. (**B**) Absolute pressure when the two slides are in glass. Since graphs are displayed with an identical scale, glass transmits less power than polystyrene.

**Figure A4:**
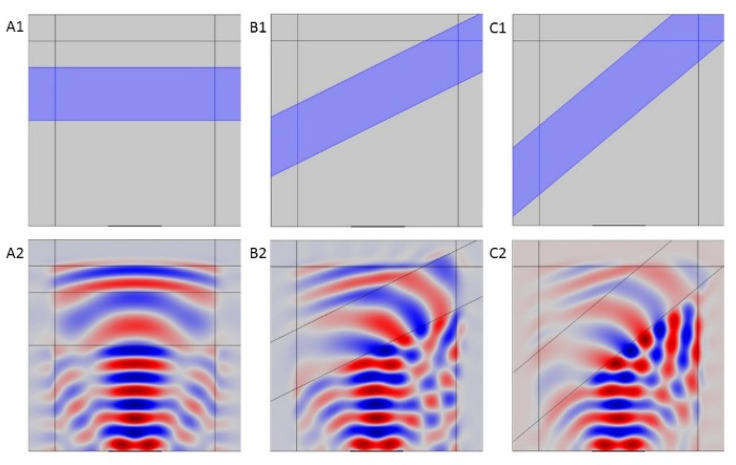
(**A1**) Simulation configuration with material 2 in blue parallel (0°) to the transducer surface. (**A2**) Propagation of an ultrasonic wave through the two materials for a normal incidence (0°). (**B1**) Simulation configuration with material 2 in blue with a 26.5° angle with the transducer surface (**B2**) Propagation of an ultrasonic wave through the two materials for at the intromission angle (26.5°) with the transducer surface. (**C1**) Simulation configuration with material 2 in blue with a 40° angle with the transducer surface (**C2**) Propagation of an ultrasonic wave through the two materials for an angle above the critical angle (40°) with the transducer.

The setup’s suitability for photoacoustic microscopy (PAM) will be investigated in a follow-up research project. When used complementary to light microscopy, PAM provides optical absorption contrast, after the cell chromophores convert light into ultrasounds. In common laboratory prototypes of PAM, optical-acoustic combiners are used with transducers mounted perpendicularly to the light path [31]; or the samples are in a water bath environment. Authors [32] designed such optical-acoustic combiner as optically transparent, but acoustically reflective by collecting ultrasounds signals from the glass-liquid interface at 23° to 45° incidence angles. On the contrary, our novel setup will be tested to detect photoacoustic signal detection at acoustically transmitting mode, as indicated by transducer intromission angle (Figure A4). By eliminating the combiner prisms, more space between the objective and the sample will be available for optical illumination with a high NA objective. In our setup, the photoacoustic signal would originate within the sample fluid layer 3 (Figure A1), then the ultrasonic waves **p**_c_ and **p**_d_ would propagate in ±**z** directions. All wave components generated at each interface of 5 layers, described as **p**_b_, **p**_a_, **p**_e_, **p**_f_, **p**_t_, **p**_i_, and **p**_r_ will produce contributions on transducer’s detected signal. The ultrasounds emitted from the chromophore, have a wide range of angles, so while all the incident waves with an angle above the critical (26.5°) are reflected, those with more acute angles will be transmitted. The amplitude of the transmitted signal is a function depending, amongst others, on the distance between the two slides, noted L. The modifications of the L parameter will be introduced during ex vivo PAM measurements. As described in the simulation part, an interference pattern occurs when the incident and reflected beams meet (Figure A2). Using polystyrene slides, the changes in L have less impact on the pressure variation within the sample, contrary to glass slides (Figure A3).

## 5. Conclusions

We propose a novel and unique setup for studies on cells and GUVs sonoporation between a microscopic slide and a microslide, with a predetermined pressure inside the sample region and in very small volumes. The analytical analysis resolution revealed the geometrical limits to respect, such as the incidence angle. Numerical simulations showed that the pressure inside the medium containing the biological objects was sufficient for sonoporation, and experiments confirmed this assessment. Glass was chosen to allow more reproducible results. This setup allows reproducible experiments because of the controlled position of the sample with respect to the transducer. The small (10 µL to 30 µL) volume necessary for experiments enables GUVs to be used. GUVs being a simple model for the cell membrane, analysis of the physic or chemical mechanisms implied in the membrane sonoporation process will be more easily approached. In this small volume, there is no need for a magnetic stirring to get a homogeneous exposure of the whole sample, which implies that the exposure time of GUVs and cells to ultrasound-activated microbubbles can be precisely known. Thanks to the simulations, there is complete knowledge of the acoustic pressure field in the chamber of insonification. This setup is a new and perfectly adequate device for the precise study of the sonoporation mechanisms. The device’s future applications in photoacoustic research are being considered.

## Figures and Tables

**Table 1:** Differences and similitudes between regular setups for cell and microbubbles exposure to US and the newly designed setup characterized here.

Medium # in a Setup	(1)	(2)	(3)	(4)	(5)
Water-bath setup	Cell media			Water bath support	Air
Clinicell™ setup	water	Clinicell™ material (flexible)	Cell media uncontrolled thickness	Clinicell™ material (flexible)	Fluid immersion
Proposed setup	water	Glass/PS (rigid)	Cell media Controlled thickness	Glass/PS (rigid)	Air

**Table 2:** The differences between a flat transducer and a focalized transducer. Results achieved using the developed numerical model.

	Focal Spot			Isosurfaces (mm²)		
Transducer	Focal Distance (mm)	Length (mm)	Width (mm)	5%	15%	25%
Flat	60	60	9	2.1	8.6	63.6
Focused	60	30	1.7	0.4	1.3	2.5

## Data Availability

Data that support findings of this study are available upon the request from the authors.

## Supplementary Materials

The following are available online at https://www.mdpi.com/article/10.3390/pharmaceutics13060847/s1, Video S1: Microbubbles oscillatory response to untrasounds recorded under the novel set-up.

## Appendix A

### Appendix A.1. Analytical Description

The analytical description of the pressure waves is the product of two terms. The first is the complex amplitude, and the second is the propagation term that takes both temporal and spatial propagation into account. The +**z** direction is defined as the wave propagation direction. It should be noted here that the frequency remains the same regardless of the media, so ω_1_ = ω_2_ = ... = ω_5_ = ω. Thus, the incident wave, the one that propagates in the +**z** direction, is described by: **p_i_** = **P_i_** e ^(**j** (ωt − **k**^_1_**^z^**^))^ and the transmitted wave, to the fifth medium, has a similar form **p_t_** = **P_t_** e^(**j** (ωt − **k**^_5_**^x^**^))^. The reflected wave **p_r_** = **P_r_** e^(**j** (ωt + **k**^**_1_^z^**^))^ propagates in the opposite direction (−**z**) [33]. At each interface, the ultrasound wave will be split into a reflected one and a transmitted one. All reflected waves in a layer have the same spatial and frequential component, so the contribution of all the waves in the complex amplitude part can be regrouped [34]. **p_a_**, **p_c_**, and **p_e_** are defined as the sum of the waves propagating through media, respectively, 2, 3, and 4 in the +**z** direction, their individual amplitudes are summed and regrouped as the complex amplitudes **A**, **C**, and **E**. Thus, **p_a_** can be written as **p_a_** = **A** e^(**j** (ω t − **k**^**_2_** ^z))^ and by extension **p_c_** = **C** e^(**j** (ω t − **k**^**_3_^z^**^))^ and **p_e_** = **E** e^(**j** (ω t − **k**^**_4_^z^**^))^. Sum of waves propagating in the –**z** in medium 2 (respectively 3 and 4) are grouped similarly as **p_b_** = **B** e^(**j** (ω t + **k**^**_2_^z^**^))^ (respectively **p_d_** = **D** e^(**j** (ω t + **k**^**_3_^z^**^))^ and **p_f_** = **F** e^(**j** (ω t + **k**^**_4_^z^**^))^). Continuity of the total energy at the interfaces combined with algebraic manipulation gives the reflection and transmission coefficients. However, what is really of interest for us is the pressure experienced by the cells and microbubbles located in the third medium. The region of interest is medium 3 and the pressure inside: P_cells_ = Re (**p_c_** + **p_d_**) = Re (**C** e^(**j** (ω t − k^**_3_^z^**^))^ + **D** e^(**j** (ω t + **k**^**_3_^z^**^))^). There is no analytical solution to determine the values of the **C** and **D** complex amplitudes. To visualize better the actual acoustic field, a numerical model of the new setup was created in: The acoustic modulus of COMSOL™, which solves the US propagation equation in the frequency domain. Often the simple transmission from one fluid to another is considered. Similarly, to the Clinicell™ setup, two solid layers separate two or three fluid layers. However, the modification of the L parameter (distance between the two slides) modifies the profile of the peak negative pressure at different distances within medium 3, containing the cells, GUVs and MB.

### Appendix A.3. Intromission Angle

An interesting feature of interface arises when r_1_ < r_2_ and c_2_ < c_1_ or r_1_ > r_2_ and c_2_ > c_1_. Under these conditions an “angle of intromission” exists and can be calculated using Equation (A1):

sin θ_1_= ((1 − (r_1_ ⁄ r_2_)^2^) / (1 − (ρ_1_ ⁄ ρ_2_)^2^))^1/2^ (A1)

At this precise angle of intromission, there is complete transmission and no reflection. Considering two materials with the following acoustic characteristic properties, ρ_1_ = 1000 kg/m^3^, ρ_2_ = 250 kg/m^3^, c_1_ = 1000 m/s, c_2_ = 2000 m/s, intromission angle here is 26.5° and critical angle 30° (Figure 4). A simulation with the same parameters confirmed the analytical results. A planar wave propagating in the first medium was simulated to test COMSOL ability to deal with refraction, intromission, and critical angles. It encounters an inclined (0°, 26.5° and 40°) obstacle materialized by the black lines. The obstacle is composed of the second medium. The results of the simulations corroborate the analytical analysis done above: Most of the wave is transmitted with a normal incidence, a greater portion goes through the interface at the intromission angle, and most of the beam is reflected above the critical angle. The intensity of the transmitted beam varies according to the incidence angle. The transmitted amplitude for an angle of 26.5° is higher than for normal incidence, which is coherent with the existence of an intromission angle with these parameters. At 40°, most of the beam is reflected since this value is above the critical angle, which was calculated at 30°, for these conditions.
